# Optimizing management of ruxolitinib in patients with myelofibrosis: the need for individualized dosing

**DOI:** 10.1186/1756-8722-6-79

**Published:** 2013-10-22

**Authors:** Ruben A Mesa, Jorge Cortes

**Affiliations:** 1 Division of Hematology & Medical Oncology, Mayo Clinic Cancer Center, 13400 E. Shea Blvd, Scottsdale, AZ 85259, USA; 2 Department of Leukemia, Division of Cancer Medicine, The University of Texas MD Anderson Cancer Center, 1515 Holcombe Boulevard, Houston, TX, USA

**Keywords:** Anemia, COMFORT-I, Dosing, JAK inhibitor, Myelofibrosis, Ruxolitinib, Thrombocytopenia

## Abstract

Ruxolitinib, an oral JAK1 and JAK2 inhibitor, is approved in the US for patients with intermediate or high-risk myelofibrosis (MF), a chronic neoplasm associated with aberrant myeloproliferation, progressive bone marrow fibrosis, splenomegaly, and burdensome symptoms. Phase III clinical studies have shown that ruxolitinib reduces splenomegaly and alleviates MF-related symptoms, with concomitant improvements in quality of life measures, for the overwhelming majority of treated patients. In addition, ruxolitinib provided an overall survival advantage as compared with either placebo or what was previously considered best available therapy in the two phase III studies. The most common adverse events with ruxolitinib treatment include dose-dependent anemia and thrombocytopenia, which are expected based on its mechanism of action. Experience from the phase III studies shows that these hematologic events can be managed effectively with dose modifications, temporary treatment interruptions, as well as red blood cell transfusions in the case of anemia and, importantly, are rarely cause for permanent treatment discontinuation. This review summarizes data supporting appropriate individualized patient management through careful monitoring of blood counts and dose titration as needed in order to maximize treatment benefit.

## Introduction

Myelofibrosis (MF), a Philadelphia chromosome-negative myeloproliferative neoplasm, is characterized by progressive bone marrow fibrosis and ineffective hematopoiesis [1,2]. Clinical presentation may include splenomegaly, anemia, and multiple burdensome chronic symptoms such as night sweats, pruritus, early satiety, abdominal pain, left subcostal pain, bone pain, profound fatigue (irrespective of presence or degree of concomitant anemia), and cachexia [3,4]. Many of these symptoms appear to be associated with a pro-inflammatory state typical for patients with MF [5], which is manifest by excessive levels of circulating cytokines such as interleukin-6 and tumor necrosis factor-α [6,7]. The molecular pathobiology of MF is characterized by dysregulation of Janus kinase (JAK)/signal transducer and activator of transcription (STAT) signaling networks [8,9], which have crucial roles in cytokine- and growth factor-mediated regulation of cellular responses, including normal hematopoiesis and inflammation [10,11]. Specifically, overactivation of JAK2 plays a role in malignant myeloproliferation, whereas aberrant JAK1 signaling contributes to many of the additional clinical and laboratory characteristics of the disease, including the debilitating symptoms associated with the pro-inflammatory state [10,12].

Before the advent of JAK inhibitors as targeted therapy, available options for the treatment of common clinical manifestations of MF, such as splenomegaly and debilitating symptoms, generally had limited, nonlasting efficacy and/or were poorly tolerated [13,14]. Ruxolitinib, an oral JAK1/JAK2 inhibitor [15] (formerly INCB018424; Incyte Corporation, Wilmington, DE, USA), is approved in the US for the treatment of patients with intermediate or high-risk MF. Outside the US, ruxolitinib is approved for the treatment of MF in 42 countries worldwide. Two phase III studies in patients with MF and platelet counts of at least 100 × 10^9^/L at baseline (the Controlled Myelofibrosis Study with Oral JAK Inhibitor Treatment [COMFORT]-I conducted in the US, Canada, and Australia, and COMFORT-II conducted in Europe) demonstrated that ruxolitinib significantly decreased spleen size, reduced MF-related symptom burden, and improved quality of life measures compared with placebo (COMFORT-I) and what at the time was considered best available therapy (BAT; COMFORT-II) [16,17]. Clinically meaningful improvements in spleen size and symptoms were also observed in an ongoing phase II study in patients with MF and baseline platelet counts of 50 × 10^9^/L to <100 × 10^9^/L [18]. Long-term data emerging from the COMFORT trials further suggest that MF patients treated with ruxolitinib have a survival advantage over those who were randomized to placebo or BAT [19,20].

Because thrombopoietin and erythropoietin signal through JAK2 [8], inhibition of JAK2 with ruxolitinib treatment is associated with dose-dependent thrombocytopenia and anemia [16,17]. In the COMFORT studies, cytopenias were managed effectively by dose adjustments and treatment interruptions or, in some instances of anemia, with red blood cell (RBC) transfusions [16,17]. As a result, only 1 patient in the ruxolitinib group discontinued therapy for anemia and 1 discontinued for thrombocytopenia at the time of the primary analysis in COMFORT-I [16]. In COMFORT-II, no patient discontinued ruxolitinib therapy for anemia and 1 discontinued for thrombocytopenia [17].

This review summarizes lessons learned from the COMFORT-I trial and from our own clinical experience, indicating that dose-related cytopenias may occur, as expected, during the course of therapy and that persistent individualized patient management, especially early during treatment, can ensure maximum treatment benefit when this medicine is used in appropriate patients with MF.

### Design and participants of the COMFORT-I study

COMFORT-I [16] is a randomized, double-blind, placebo-controlled, phase III trial in patients with intermediate-2 or high-risk MF (including primary, post-essential thrombocythemia, and post-polycythemia vera MF) with a baseline platelet count of at least 100 × 10^9^/L. Of the 309 study participants, 155 were randomized to receive ruxolitinib and 154 were randomized to receive placebo. The starting dose of the blinded study treatment was based on each patient’s platelet count at baseline, ie, 15 mg twice daily (BID) for patients with a platelet count of 100 to 200 × 10^9^/L and 20 mg BID for those with a platelet count >200 × 10^9^/L. The primary endpoint was the percentage of patients with at least a 35% reduction from baseline in spleen volume (as assessed by abdominal imaging) at week 24. A key secondary endpoint was the percentage of patients with a 50% or greater improvement in Total Symptom Score (TSS, comprising individual scores for night sweats, pruritus, bone or muscle pain, abdominal discomfort, pain under left ribs, and early satiety) assessed with the modified Myelofibrosis Symptom Assessment Form (MFSAF), version 2.0.

### Management of treatment-related cytopenias in COMFORT-I

#### ***Onset of anemia and thrombocytopenia***

**Figure 1:**
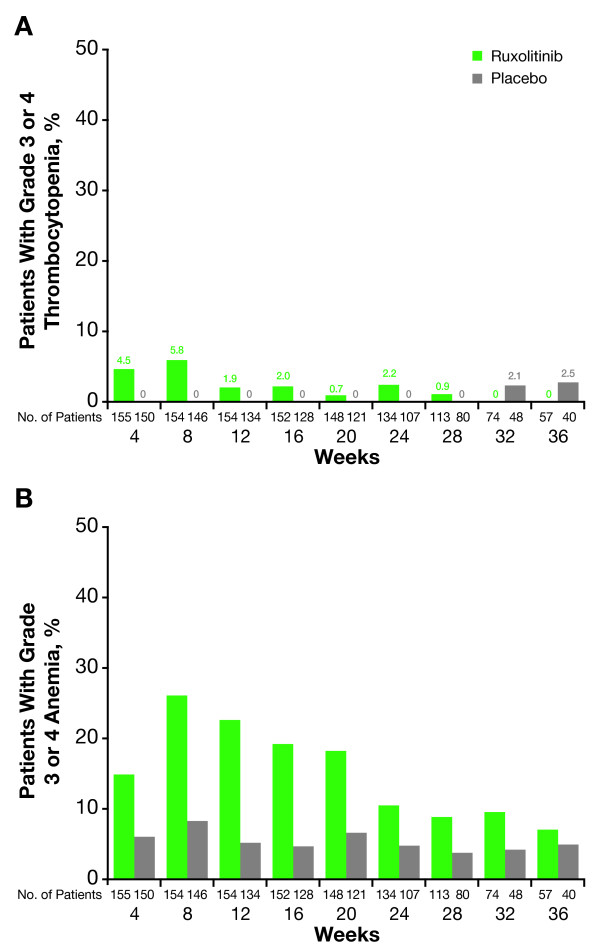
**Rates of grade ≥3 thrombocytopenia and anemia in COMFORT-I.** (From Verstovsek, et al. A double-blind, placebo-controlled trial of ruxolitinib for myelofibrosis [16]. © 2012 Massachusetts Medical Society. Reprinted with permission from the Massachusetts Medical Society). Shown are mean percentages of **(A)** grade ≥3 thrombocytopenia and **(B)** grade ≥3 anemia per month over time.

**Figure 2:**
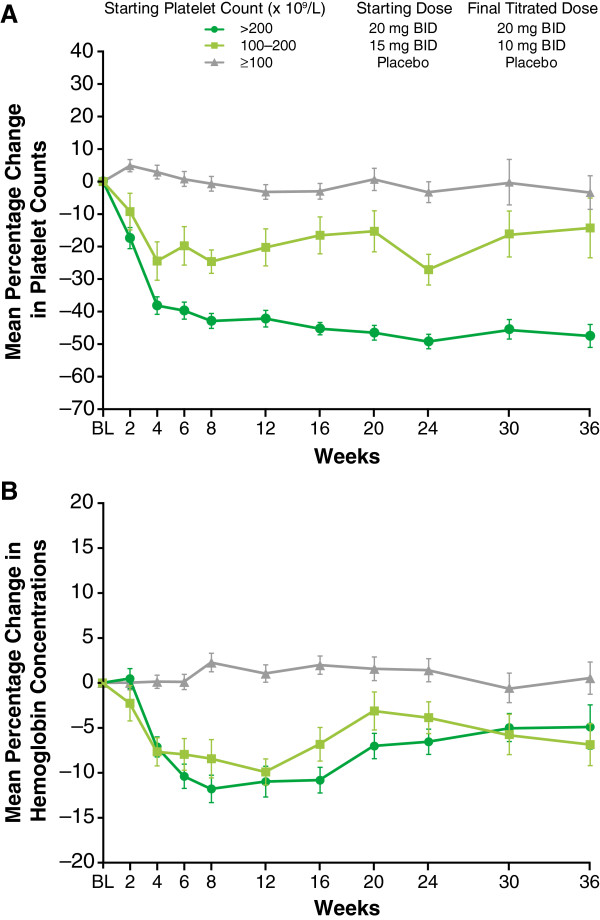
**Changes in platelet count and hemoglobin in COMFORT-I.** (Reprinted from Verstovsek S, et al. Management of cytopenias in patients with myelofibrosis treated with ruxolitinib and effect of dose modifications on efficacy outcomes. Onco Targets Ther [published by Dove Press] [22]). Shown are mean percentage changes from baseline (BL) with standard errors in **(A)** platelet count and **(B)** hemoglobin over time. Final titrated dose was defined as the average daily dose during weeks 21–24. BID, twice a day.

Anemia and thrombocytopenia were the most common adverse events associated with ruxolitinib treatment and typically occurred early in the course of therapy [16,17,21]. As shown in Figure 1A and 1B, grade 3 or 4 anemia and thrombocytopenia were greatest in the first 8–12 weeks of treatment [16]. Similarly, hemoglobin levels and platelet counts decreased during the same time frame (Figure 2A and 2B) [22].

#### ***Management of cytopenias***

Cytopenias in COMFORT-I were managed successfully by the use of dose adjustments and RBC transfusions (for anemia). This management strategy included mandatory dose reductions at moderate levels of thrombocytopenia to prevent further decreases in platelet counts and to minimize the need for temporary or permanent treatment discontinuation. Doses could be increased step-wise once blood counts had recovered. This strategy is the basis for the current dosing recommendations for patients with platelet counts ≥100 × 10^9^/L (Table 1).

**Table 1:** **Ruxolitinib dose modifications recommended for MF patients with starting platelet count of at least 100 × 10**^**9**^/L*

	**Dose at time of decline in platelet count**					**Maximum dose based on platelet count after prior treatment interruption or dose reduction**
	**25 mg BID**	**20 mg BID**	**15 mg BID**	**10 mg BID**	**5 mg BID**	
**Current platelet count**	**New dose to be used**					
≥125 × 10^9^/L	No change	No change	No change	No change	No change	20 mg BID
100 to <125 × 10^9^/L	20 mg BID	15 mg BID	No change	No change	No change	15 mg BID
75 to <100 × 10^9^/L	10 mg BID	10 mg BID	10 mg BID	No change	No change	10 mg BID for 2 weeks; if stable, may increase to 15 mg BID
50 to <75 × 10^9^/L	5 mg BID	5 mg BID	5 mg BID	5 mg BID	No change	5 mg BID for 2 weeks; if stable, may increase to 10 mg BID
<50 × 10^9^/L	Hold	Hold	Hold	Hold	Hold	Continue holding

*Starting ruxolitinib doses of 15 mg BID for patients with platelet counts of 100 to 200 × 10^9^/L and 20 mg BID for those with a platelet count >200 × 10^9^/L. Recommended dose modifications based on US prescribing information.

For insufficient response, doses may be increased in 5-mg BID increments to a maximum of 25 mg BID, provided that platelet and neutrophil counts are adequate.

BID, twice daily; MF, myelofibrosis.

Data from Jakafi prescribing information (Incyte Corporation, June 2013).

See full prescribing information for a complete description of FDA-approved dosing of ruxolitinib in patients with intermediate or high-risk MF.

Of 155 patients randomized to ruxolitinib, 56% required dose reductions; however, more than half of those patients (49/87) had only a single dose reduction [22]. Most dose reductions occurred during the first 8 to 12 weeks, because decreases in platelet counts primarily occurred during this time period. An analysis of the average daily doses in the ruxolitinib arm of COMFORT-I from week 21 to week 24 (final titrated doses) showed that approximately 60% of patients with baseline platelet counts of 100 to 200 × 10^9^/L and more than 95% of those with counts >200 × 10^9^/L attained doses of 10 mg BID or greater; the median doses in these groups were approximately 10 and 20 mg BID, respectively [22].

Implementation of mandatory dose reductions and treatment interruptions was associated with stabilization of mean platelet counts after the first 8 to 12 weeks of treatment (Figure 2A) [22]. Mean hemoglobin levels recovered to near baseline levels (Figure 2B) after reaching a nadir of 95 g/L at approximately 8 to 12 weeks of treatment, and RBC transfusion requirements followed a similar trend [16,22]. This time course of mean hemoglobin values observed in COMFORT-I was confirmed in COMFORT-II. After decreasing from a baseline value of 109.3 g/L to a nadir of 94.1 g/L approximately 12 weeks after treatment initiation, mean hemoglobin levels in the ruxolitinib arm of COMFORT-II reached a steady-state value of 101.8 g/L by week 24 [17]. Monthly rates of grade 3 or 4 anemia and thrombocytopenia in COMFORT-I decreased over time to levels similar to those observed in the placebo group (Figure 1) [16]. In addition, grade 3 or 4 episodes of bleeding with ruxolitinib were uncommon (2.6% and 1.3%, respectively) and occurred at rates similar to those with placebo (2.0% and 1.3%, respectively), an indication that the mandatory dose reductions and treatment interruptions allowed for effective management of thrombocytopenia [16].

#### ***Effect of ruxolitinib dose modifications on efficacy***

**Figure 3:**
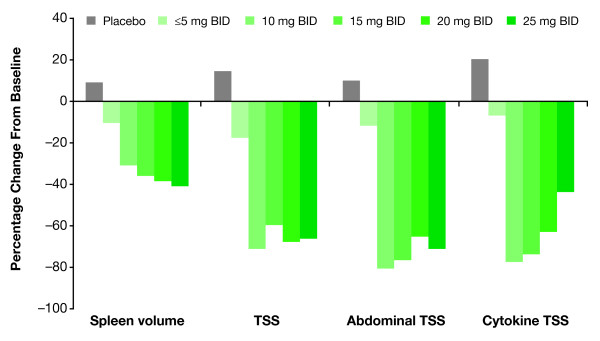
**Changes in spleen volume and symptom scores in COMFORT-I by final titrated ruxolitinib dose.** (Adapted from Verstovsek S, et al. Management of cytopenias in patients with myelofibrosis treated with ruxolitinib and effect of dose modifications on efficacy outcomes. Onco Targets Ther [published by Dove Press] [22]). Shown are median percentage changes from baseline to week 24 in spleen volume, total symptom score (TSS), abdominal TSS, and cytokine TSS by final titrated ruxolitinib dose (average dose during weeks 21–24). Abdominal TSS includes scores for abdominal discomfort, pain under the ribs on the left side, and early satiety. Cytokine TSS includes scores for night sweats, pruritus, and muscle/bone pain. BID, twice daily.

Although these data illustrate that ruxolitinib-associated cytopenias can be managed effectively with dose modifications, it is also important to understand how such dose modifications may affect the efficacy of ruxolitinib therapy. Final titrated doses of ruxolitinib ≥10 mg BID (calculated as average daily dose during weeks 21–24) were associated with clinically meaningful reductions in spleen volume and improvement in MF-related symptoms from baseline to week 24 (Figure 3) [22]. The median reduction in spleen volume at a final titrated dose of 10 mg BID (30.8%) [22] was similar to that observed in the overall ruxolitinib group (33.0%) [16], with slightly greater reductions at higher titrated doses. Median reductions in both cytokine and abdominal TSS showed similar symptom improvements at doses of 10 mg BID or higher [22].

### Ruxolitinib use in clinical practice: lessons from COMFORT-I

Treatment-related cytopenias in MF patients receiving ruxolitinib are dose-dependent and, given the mechanism of action of ruxolitinib, they are to be expected. However, in patients with similar blood cell counts at baseline, the risk of new-onset or worsening cytopenias at a given dose of ruxolitinib may vary widely. This is likely because of the considerable heterogeneity of clinical characteristics among patients—even if they belong to the same risk category—and the consequent differences in the clinical impact of ruxolitinib. Therefore, dose adjustments, including their timing, need to be tailored to each patient and be accompanied by careful and appropriate monitoring of both clinical manifestations (symptoms and splenomegaly) and hematologic parameters. In particular, aggressive dose increases during the first 3 months of therapy should only be considered if a patient consistently maintains adequate platelet counts.

The need for careful patient monitoring and dose optimization in MF patients receiving treatment is not unique to ruxolitinib. Similar examples in hematology/oncology include azacitidine and lenalidomide in patients with myelodysplastic syndrome. Azacitidine may cause anemia, neutropenia and thrombocytopenia [23], and complete blood counts (CBCs) before each treatment cycle (of 28 days) and as needed are mandated per prescribing information (Celgene Corporation, May 2012) to monitor response and toxicity. Lenalidomide is commonly associated with grade 3 or 4 thrombocytopenia or neutropenia [24], and dose reductions or treatment interruptions (depending on specific platelet and absolute neutrophil counts) are recommended [25].

To manage cytopenias with ruxolitinib therapy in clinical practice, starting doses should be chosen based on platelet count and subsequently adjusted based on CBCs, which need to be monitored every 2 to 4 weeks, or as clinically indicated, during the first 8 to 12 weeks of therapy or until a stable dose has been reached. Dose titration in patients with platelet counts of at least 100 × 10^9^/L should follow the guidelines outlined in Table 1, in accordance with US prescribing information. In addition, dose adjustments and RBC transfusions may be considered for the management of anemia.

Similar to the practice in COMFORT-I, starting doses in COMFORT-II were based on platelet count, and dose modifications were mandated in cases of developing thrombocytopenia or neutropenia [17]. Dosing recommendations outside the US essentially follow the practice in COMFORT-II: starting doses of 15 and 20 mg BID are recommended for patients with platelet counts of 100 to 200 × 10^9^/L and those with >200 × 10^9^/L, respectively, with close monitoring of blood counts and modifications in dosing based on platelet or neutrophil counts as well as clinical response.

Because COMFORT-I and COMFORT-II did not include patients with baseline platelet counts lower than 100 × 10^9^/L [16], an optimized ruxolitinib dosing strategy for patients with intermediate or high-risk MF and baseline platelet counts of 50 to 100 × 10^9^/L is being evaluated in an open-label, phase II study [18]. Preliminary results supported an update of the US prescribing information in June 2013 to include specific recommendations for the management of patients with a platelet count of at least 50 × 10^9^/L but less than 100 × 10^9^/L at the start of therapy. Key features of these recommendations are a starting dose of 5 mg BID and subsequent dose modifications based on efficacy and changes in platelet count, with a maximum dose of 10 mg BID (Table 2).

**Table 2:** **Ruxolitinib dose modifications recommended for MF patients with a starting platelet count of at least 50 × 10**^**9**^**/L but less than 100 × 10**^**9**^/L*

**Current platelet count**	**Dosing recommendation**
<25 × 10^9^/L	Interrupt treatment
25 to <35 × 10^9^/L with <20% decrease during the prior 4 weeks	Decrease dose by 5 mg QD or maintain the current dose if it is 5 mg QD
25 to <35 × 10^9^/L with ≥20% decrease during prior 4 weeks	Decrease dose by 5 mg BID or use 5 mg QD if the current dose is 5 mg BID or QD
≥40 × 10^9^/L with ≤20% decrease during prior 4 weeks, ANC >1 × 10^9^/L, and no dose reductions or treatment interruptions for AE or hematologic toxicity during the prior 4 weeks	Increase dose by increments of 5 mg QD to a maximum of 10 mg BID if response is insufficient

*Starting ruxolitinib dose of 5 mg BID. Recommended dose modifications based on US prescribing information.

AE, adverse event; ANC, absolute neutrophil count; BID, twice daily; FDA, US Food and Drug Administration; MF, myelofibrosis; QD, once daily.

Data from Jakafi prescribing information (Incyte Corporation, June 2013).

See full prescribing information for a complete description of FDA-approved dosing of ruxolitinib in patients with intermediate or high-risk MF.

An important lesson from COMFORT-I is that dose adjustments for cytopenias generally do not affect efficacy. The vast majority of patients in COMFORT-I attained doses of 10 mg BID or higher after titration, and these titrated dose levels have been associated with clinically meaningful reductions in spleen volume and symptom burden [22]. Thus, patients in clinical practice, even those with low platelet counts at the beginning of therapy, may achieve stable doses that are clinically effective. This conclusion is supported by COMFORT-I data and consistent with the preliminary results of the ongoing phase II study of ruxolitinib in patients with baseline platelet counts of 50 to 100 × 10^9^/L [18]. In that study, dose titration of ruxolitinib from a starting dose of 5 mg BID to final titrated doses of 10 mg BID or higher was associated with stable mean hemoglobin levels over time as well as clinically meaningful symptom relief and spleen volume reduction [18]. These findings further underscore the importance of initial dose optimization for ensuring continuity of treatment benefit by minimizing dose-dependent hematologic toxicity while preserving efficacy. Thus, onset or worsening of cytopenias should not lead to immediate treatment discontinuation, but rather to careful management of these events.

## Conclusions

Ruxolitinib is associated with dose-dependent risks of thrombocytopenia and anemia in MF patients, as expected from its mechanism of action; these treatment-related cytopenias do not indicate a worsening of the underlying disease. Dose adjustments can be effective in the management of these cytopenias without compromising efficacy. Persistent monitoring and management, particularly over the first 8 to 12 weeks of ruxolitinib therapy, are necessary to ensure that patients attain stable doses that are both safe and provide maximum long-term benefit.

## Abbreviations

BAT: Best available therapy; BID: Twice daily; CBC: Complete blood count; COMFORT: Controlled Myelofibrosis Study with Oral JAK Inhibitor Treatment; JAK: Janus kinase; MF: Myelofibrosis; MFSAF: Myelofibrosis symptom assessment form; RBC: Red blood cell; TSS: Total symptom score.

## Competing interests

RAM has received research funding from Incyte Corporation, Gilead, NS Pharma, Genentech, and Lilly. JC is a consultant for and has received research funding from Incyte Corporation and Sanofi.

## Authors’ contributions

Both authors contributed to the conception and design of this review, participated in the drafting of the manuscript, and approved its final version.

## Authors’ information

RAM is Deputy Director of the Mayo Clinic Cancer Center and the Chair of its Division of Hematology & Medical Oncology in Scottsdale, AZ. JC is Deputy Chair of the Department of Leukemia at the Division of Cancer Medicine of the University of Texas MD Anderson Cancer Center in Houston, TX.
